# BATF Potentially Mediates Negative Regulation of PD-1/PD-Ls Pathway on T Cell Functions in *Mycobacterium tuberculosis* Infection

**DOI:** 10.3389/fimmu.2019.02430

**Published:** 2019-10-15

**Authors:** Qianqian Liu, Qinfang Ou, Lei Shen, Chao Qiu, Bingyan Zhang, Wenhong Zhang, Lingyun Shao, Yan Gao, Zheng W. Chen

**Affiliations:** ^1^Department of Infectious Diseases, Huashan Hospital, Fudan University, Shanghai, China; ^2^Department of Pulmonary Diseases, Wuxi Infectious Diseases Hospital, Wuxi, China; ^3^Department of Thoracic Surgery, Shanghai Pulmonary Hospital, Shanghai, China; ^4^Institutes of Biomedical Sciences, Fudan University, Shanghai, China; ^5^Key Laboratory of Medical Molecular Virology, Ministry of Education and Health, Institutes of Biomedical Science, Shanghai Medical College, Fudan University, Shanghai, China; ^6^Department of Microbiology and Immunology, Center for Primate Biomedical Research, University of Illinois College of Medicine, Chicago, IL, United States

**Keywords:** BATF, tuberculosis, PD-1/PD-Ls pathway, T cell function, immune response

## Abstract

**Background:** Previously, we have found that blockade of PD-1/PD-Ls pathway could enhance CD4^+^ T cells-mediated protective immunity in patients with active tuberculosis (ATB). However, the mechanism of PD-1/PD-Ls pathway involved in negative regulation of anti-TB immunity has been still unclear. Recently, the study of human immunodeficiency virus (HIV) infection demonstrated that PD-1 could induce the expression of basic leucine zipper ATF-like transcription factor (BATF) to inhibit CD8^+^ T cell function. While the mechanism of immune regulation of BATF in *Mycobacterium tuberculosis* (*M. tb*) infection has not yet been elucidated.

**Methods:** We enrolled 104 participants including ATB patients (*n* = 66), latent tuberculosis infection (LTBI) (*n* = 16) and healthy control (HC) (*n* = 22). The expressions of BATF in peripheral blood CD4^+^ and CD8^+^ T cells from enrolled subjects were determined using flow cytometry. Intervention with PD-1/PD-Ls pathway was performed by using blocking antibodies or human PD-L1 fusion protein. Silencing BATF in peripheral blood mononuclear cells (PBMCs) by electroporation with siRNA. Real-time quantitative PCR, CFSE dilution assay and enzyme linked immunosorbent assay (ELISA) were employed to test T cell functions after BATF knockdown.

**Results:** The percentages of BATF^+^CD4^+^ (*P* = 0.0003 and *P* < 0.0001, respectively) and BATF^+^CD8^+^ (*P* = 0.0003 and *P* = 0.0003, respectively) cells were significantly increased in ATB patients compared with LTBI and HC. BATF-expressing PD-1^+^ T cells in CD4^+^ and CD8^+^ T cells were much higher in ATB group than those in LTBI group (*P* = 0.0426 and 0.0104, respectively) and HC group (*P* = 0.0133 and 0.0340, respectively). There was a positive correlation between BATF expression and PD-1 expression in ATB patients (for CD4^+^ T cells, *r* = 0.6761, *P* = 0.0158; for CD8^+^ T cells, *r* = 0.6104, *P* = 0.0350). BATF knockdown could enhance IL-2 and IFN-γ secretions (*P* = 0.0485 and 0.0473, respectively) and CD4^+^ T cells proliferation (*P* = 0.0041) *in vitro*.

**Conclusions:** In the context of tuberculosis, BATF mediates negative regulation of PD-1/PD-Ls pathway on T cell functions. BATF knockdown can improve cytokine secretion and cells proliferation *in vitro*.

## Introduction

Tuberculosis (TB), caused by *Mycobacterium tuberculosis* (*M. tb*), still remains one of the global public health problem. WHO reported an estimated 10.4 million new TB cases worldwide in 2016 and 1.67 million deaths due to TB (1). *M. tb*, as an intracellular pathogen, interacting with host immune system determines the outcome of infection (2–4). Therefore, immune response plays a key role in host defense against *M. tb* infection.

Inhibitory receptor programmed cell death protein 1, also known as PD-1, is a member of CD28 family, which plays an important role in immunosuppressive signaling by binding to its ligands PD-L1 or PD-L2 (5, 6). Our previous study found that the expression of PD-1 and its ligands PD-L1 or PD-L2 on peripheral blood immune cells were elevated in patients with active tuberculosis (ATB) (7). In addition, blockade of PD-1/PD-Ls pathway could improve not only *M. tb*-specific CD4^+^ T cell-mediated protective immunity but also phagocytosis of macrophages in TB infection (7, 8). After 6 months of effective anti-TB therapy, we observed a significant decrease in PD-1 expression on effector T cells and responder T cells (CD4^+^CD25^−^ subsets) from 6 ATB patients (9). However, the mechanism of PD-1/PD-Ls pathway involved in negative regulation of anti-TB immunity has been still unclear (7, 8, 10). Recently, it has been confirmed in study of chronic human immunodeficiency virus (HIV) infection that PD-1/PD-Ls pathway function to inhibit T cells by inducing the expression of basic leucine zipper ATF-like transcription factor (BATF) in CD8^+^ T cells in addition to reducing T cell receptor (TCR) signaling (11).

BATF, a member of the activator protein 1 (AP-1) family, has a similar structure with AP-1 composed of Jun and Fos protein, but it is lack of a conventional transcription factor activation region (12–14). It is well known that Jun/Fos heterodimer plays a positive regulatory role in cell differentiation and proliferation, whereas BATF binds to Jun protein via competition with Fos protein to form Jun/BATF heterodimer instead of Jun/Fos heterodimer binding to DNA, which leads to inhibit activation of transcription factor activation signaling and further negatively regulate of AP-1-mediated function (15). The BATF family consists of BATF, BATF2 and BATF3. Among them, BATF and BATF3 are mainly involved in the immune regulation of dendritic cells (DC), T cells and B cells, while BATF2 is mainly expressed in M1 macrophages and involved in Th1 immune response (16–18).

At present, studies on BATF in infectious diseases mainly focus on viral infections, such as epstein-barr virus (EBV) (19), HIV (11) and lymphocytic choriomeningitis virus (LCMV) (20), but few studies on bacterial infections, especially on *M. tb* infection. Surprisingly, increased expression of BATF in macrophage infected with *M. tb* was observed in a murine model (21). In a clinical cohort study, the expression of BATF2 in peripheral blood of patients with ATB in HIV-negative populations was much higher than that in patients with latent tuberculosis infection (LTBI) and healthy controls (HC), furthermore, BATF2 in peripheral blood can be used as a novel biomarker for the diagnosis of TB with an area under the curve (AUC) of 0.93–0.99 (22). However, the mechanism of immune regulation of BATF in *M. tb* infection has not yet been elucidated. Therefore, in the context of TB, we explored the role of BATF in immune regulation and whether BATF could mediate PD-1/PD-Ls pathway to play an immunosuppressive effect on *M. tb*-specific T cell functions.

## Materials and Methods

### Study Population

A total of 104 individuals were enrolled in this study, including patients with ATB (*n* = 66), population with LTBI (*n* = 16) and HC (*n* = 22). ATB patients were recruited from Wuxi Fifth People's Hospital and Zhuji People's Hospital from July 2015 to July 2017. The diagnosis of ATB was based on clinical symptoms, radiological data and identification of acid-fast bacilli in sputum. Population with LTBI and HC were recruited from the relatives of ATB patients and the volunteers of Fudan University Affiliated Huashan Hospital during the same period. This study was approved by the Ethics committee of Huashan Hospital, Fudan University. And written informed consent was obtained in accordance with the Declaration of Helsinki.

### Cell Surface and BATF Intracellular Stainings

Peripheral blood mononuclear cells (PBMCs) were freshly isolated from 10 mL EDTA anticoagulated venous blood using Ficoll density-gradient centrifugation and resuspended in RPMI-1640 medium (Invitrogen-Gibco, USA), supplemented with 10% heat-inactivated FBS (Invitrogen-Gibco, USA). To investigate the expression of BATF in peripheral blood T cells, PBMCs (1 × 10^6^) were surface stained with CD4-PB (RPA-T4, BD Pharmingen), CD8-PE-Cy7 (RPA-T8, BD Pharmingen) and PD-1-FITC (MIH4, eBioscience). Cells were then fixed and permeabilized with Cytofix/Cytoperm buffer according to the manufacturer's instruction, stained with BATF-APC (687706, R&D Systems). Stained samples were fixed with 2% paraformaldehyde (PFA), and detected on a BD FACS Aria device. Data were analyzed with FlowJo software (Tree Star Inc., USA).

### Blockade of PD-1/PD-Ls Pathway (7)

In separate experiments, freshly isolated PBMCs (1 × 10^6^ cells per well) from ATB patients were cultured in 96-well flat-bottom plates and stimulated with purified protein derivative (PPD) (25 μg/mL, Prionics AG), anti-CD28 (1 μg/mL, CD28.2, BD Pharmingen) and anti-CD49d (1 μg/mL, 9F10, BD Pharmingen) overnight. In another experiments, before the stimulation, cells were pretreated with blocking antibodies (Abs) against PD-1 (10 μg/mL, J116, eBioscience) or PD-L1 (10 μg/mL, MIH1, eBioscience) and PD-L2 (10 μg/mL, MIH18, eBioscience) for 1 h. Then, cells were collected and stained with CD4-PB, CD8-PE-Cy7 and BATF-APC. The percentage of BATF-expressing cells was analyzed using flow cytometry.

### Plate-Bound T Cell Activation Assay (23, 24)

PBMCs (1 × 10^6^ cells per well) were cultured in 96-well flat-bottom plates in the presence of either anti-CD3 (3 μg/mL, OKT3, eBioscience) alone or in combination with human PD-L1 Fc fusion protein (2 μg/mL, BPS Bioscience) in 5% CO_2_ at 37°C for 72 h, then, stimulated with PPD (25 μg/mL), anti-CD28 (1 μg/mL) and anti-CD49d (1 μg/mL) overnight. In the end, cells were treated with Trizol Reagent (Invitrogen, USA) and stored at −80°C.

### Real-Time Quantitative PCR

For quantitative PCR analysis, total RNA of PBMCs was extracted using Trizol according to manufacturer's introduction. And the concentration of total RNA was detected via Nanodrop 2000C (Thermo Scientific, USA). Then 500 ng total RNA was converted to cDNA with reverse transcription kit (Takara, Japan). The cDNA product was amplified via real-time PCR system (ABI 7500, USA) using SYBR® Premix Ex Taq^TM^ II Kit (Takara, Japan) with 40 cycles of 95°C, 5 s; and 60°C, 34 s. The primers of human GAPDH, BATF, IFN-γ, and IL-2 were synthesized by Sangon Biotech (Shanghai, China). And the primer sequences were as follows: GAPDH (forward 5′-CCCCTTCATTGACCTCAACTAC-3′, reverse 5′-GATGACAAGCTTCCCGTTCTC-3′); BATF (forward 5′-AGCGAAGACCTGGAGAAACA-3′, reverse 5′-TTCAGCACCGACGTGAAGTA-3′); IFN-γ (forward 5′-TGGCTTTTCAGCTCTGCATCGT-3′, reverse 5′-TCCACACTCTTTTGGATGCTCTGGT) and IL-2 (forward 5′-CATCCTGGTGAGTTTGGGATTC-3′, reverse 5′-TCCTGTCTTGCATTGCACTAAG-3′). Expression values of genes were estimated using 2^−ΔΔ*Ct*^ method, and GAPDH was used to normalize the BATF, IFN-γ, and IL-2 results.

### BATF Small Interfering RNA (siRNA) Knockdown in ATB Patients (11)

Inhibition of BATF expression was achieved by siRNA transfection via electroporation on a Lonza Nucleofector II Device (Lonza, Germany). Per 5 × 10^6^ PBMCs from ATB patients were resuspended in 100 μL room-temperature balanced Nucleofector®solution (Human T Cell Nucleofector®Kit, Lonza, Germany), and added with 200 nM siRNA (ON-TARGET plus Non-targeting pool or BATF ON-TARGET plus SMART pool, GE Dharmacon, USA) or 2 μg GFP Vector (Lonza, Germany). Then, cells were electroporated according to manufacturer's introduction and incubated in 1mL pre-warmed Opti-MEM medium (Invitrogen-Gibco, USA) in a 12-well plate in 5% CO_2_ at 37°C. Serum-containing medium changed 6 h post transfection. After electroporation, cells were staining with 0.4% trypan blue for cell count and viability assessment, and cell viability was usually 80%. After 24 h, transfection efficiency was evaluated with GFP protein expression of transfected cells through flow cytometry. After a 72-h incubation, transfected PBMCs were harvested for subsequent assessment of cell function or used in T cell proliferation assay.

### *In vitro* T Cell Proliferation Assay

Cell proliferation was determined by a CFSE dilution assay. Transfected PBMCs were prepared as described above and labeled with 2.5 μM pre-heated CFSE (Invitrogen, USA). CFSE-labeled PBMCs (10^5^ cells per well) were stimulated with PPD (25 μg/mL), anti-CD3 (2.5 μg/mL, BD Bioscience) and anti-CD28 (2.5 μg/mL, BD Bioscience), and cultured in 5% CO_2_ at 37°C. After 4 days, proliferated cells were stained with CD4-PB (BD Pharmingen) and detected via dilution of CFSE on a flow cytometer. Percentage of cells with diluted CFSE was determined in gated populations of total CD4^+^ T cells.

### Enzyme Linked Immunosorbent Assay (ELISA)

For analysis of cytokine secretion from transfected PBMCs, cultures were set up as described above and supernatants were harvested at 24 h after anti-CD3/anti-CD28 activation. The levels of IFN-γ and IL-2 were measured using human ELISA kits (eBioscience, USA) according to the manufacturers' instructions.

### Statistical Analysis

Data were analyzed using GraphPad Prism 6 (GraphPad, Inc., La Jolla, CA, USA). Comparisons between groups were made using unpaired *t-*tests or Mann-Whitney tests (when the variances were significantly different). The relationship between BATF expression and PD-1 expression on T cells was analyzed using Pearson correlation test. Statistical significance between negative control siRNA group and BATF siRNA group was evaluated with paired *t-*test. Significance was inferred for *P* < 0.05.

## Results

### Clinical Characteristics of Enrolled Participants

The 104 enrolled participants were divided into three groups according to the status of tuberculosis infection, including ATB group (*n* = 66), LTBI group (*n* = 16) and HC group (*n* = 22). In the ATB group, all of patients were confirmed pulmonary tuberculosis based on smear positive for acid-fast bacilli or culture positive for *M. tb* in sputum. The average age was 42.8 years, and 40 out of 66 patients were male (60.6%). About two thirds of ATB patients (*n* = 43) had received anti-TB therapy, and the treatment duration at enrollment was no more than 4 weeks. Two out of 66 (3.0%) ATB patients had a history of exposure to ATB. Sixteen LTBI individuals were all IFN-γ release assay (IGRA)-positive and had no evidence of ATB. The average age was 35.9 years, and 8 out of 16 (50%) were male. Among them, 4 (25%) had a history of exposure to ATB. In the HC group, all of them were IGRA-negative and had no evidence of ATB. The average age was 34.6 years, and 7 out of 22 (31.8%) were male. Six (27.3%) had a history of exposure to ATB. Table 1 showed the characteristics of enrolled three groups.

**Table 1:** The characteristics of the enrolled individuals.

	**ATB**	**LTBI**	**HC**	*P*-value^*^
Numbers of subjects	66	16	22	/
Average age, years (range)	42.8 (18–82)	35.9 (25–58)	34.6 (22–67)	0.0589
Gender, male/female	40/26	8/8	7/15	0.0623
History of BCG vaccination, n (%)	58 (87.9)	16 (100.0)	22 (100.0)	0.1210
History of exposure to ATB, n (%)	2 (3.0)	4 (25.0)	6 (27.3)	0.0016
Sputum AFB smear or culture positive, n (%)	66 (100.0)	/	/	/
Duration of anti-TB therapy at enrollment, average days (range)	8.1 (1–28)	/	/	/

*ATB, active tuberculosis; LTBI, latent tuberculosis infection; HC, healthy control; BCG, bacillus Calmette-Guerin; AFB, acid-fast bacilli; TB, tuberculosis*.

* *Compared among ATB, LTBI, and HC groups*.

All enrolled participants were free of HIV infection, cancer, diabetes, autoimmune diseases or other chronic infections (i.e., chronic HBV/HCV infection). Furthermore, they were free of immune-modulating treatments.

### Correlation Between Upregulation of BATF and PD-1 in the Peripheral Blood T Cells of Patients With ATB

**Figure 1:**
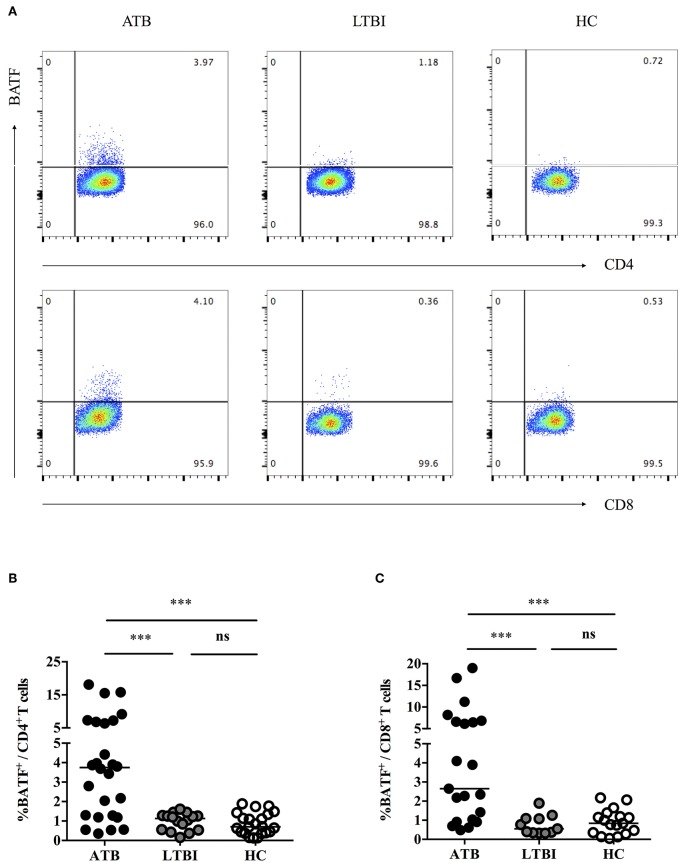
Expressions of BATF in the peripheral blood T cells among subjects with different statuses of tuberculosis infection. **(A)** Representative CD4- and CD8-gated dot plots of BATF expression. The percentages of BATF^+^ cells in CD4^+^ and CD8^+^ T cells among different populations were measured by flow cytometry. **(B)** BATF expression of CD4^+^ T cells in the peripheral blood of ATB group, LTBI group, and HC group. The horizontal lines represent the medians for each group. **(C)** BATF expression of CD8^+^ T cells in the peripheral blood of ATB group, LTBI group, and HC group. The horizontal lines represent the medians for each group. ^***^P < 0.001. ATB, active tuberculosis; LTBI, latent tuberculosis infection; HC, healthy control; ns, not significant.

**Figure 2:**
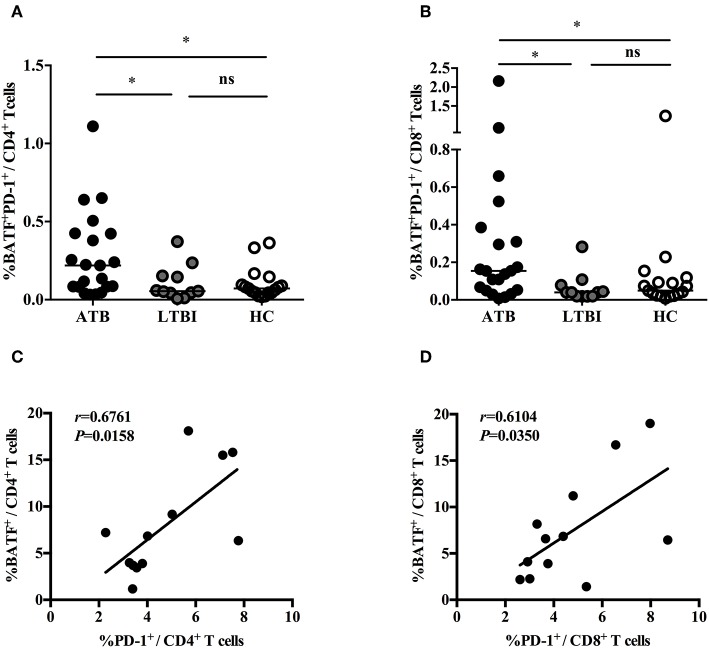
The correlations between PD-1 expression and BATF expression in T cells. **(A)** Expression of BATF in the peripheral blood PD-1-expressing CD4^+^ T cells of ATB group, LTBI group, and HC group. The horizontal lines represent the medians for each group. **(B)** Expression of BATF in the peripheral blood PD-1-expressing CD8^+^ T cells of ATB group, LTBI group, and HC group. The horizontal lines represent the medians for each group. **(C)** Correlation analysis between the expression of BATF and PD-1 in CD4^+^ T cells among ATB individuals. **(D)** Correlation analysis between the expression of BATF and PD-1 in CD8^+^ T cells among ATB individuals. ^*^P < 0.05. ATB, active tuberculosis; LTBI, latent tuberculosis infection; HC, healthy control; ns, not significant.

Previously, we demonstrated that PD-1/PD-Ls pathway inhibited *M.tb*-specific CD4^+^ T-cell-mediated protective immune response in patients with ATB (7, 9). A recent study found that the inhibitory receptor PD-1 could induce the expression of BATF to impair T cell function in HIV-infected progressive individuals (11). However, it is unknown whether BATF plays an immunosuppressive role on chronic *M. tb* infection. To explore this, first of all, we investigated BATF expressions in the peripheral blood CD4^+^ and CD8^+^ T cells of populations with different tuberculosis infection statuses using flow cytometry (gating strategy see Figures S1–S3). As expected, upregulated expression of BATF in CD4^+^ T cells was observed in patients with ATB compared with LTBI (3.75 vs. 1.13%, *P* = 0.0003) and HC (3.75 vs. 0.71%, *P* < 0.0001) (Figures 1A,B). Similarly, BATF expression of CD8^+^ T cells in ATB patients was markedly higher than that in LTBI group (2.65 vs. 0.55%, *P* = 0.0003) and HC group (2.65 vs. 0.84%, *P* = 0.0003) (Figures 1A,C). We further compared the expression of BATF in LTBI group and HC group, there was no significant difference between LTBI and HC (Figures 1B,C). We next asked whether upregulated expression of BATF was related with inhibitory receptor PD-1 in ATB patients. To determine this, we first analyzed the frequency of BATF-expressing PD-1^+^ T cells among three groups. As expected, the frequency of BATF-expressing PD-1^+^ T cells in CD4^+^ and CD8^+^ T cells were significantly higher in ATB group than those in LTBI group (0.219 vs. 0.055%, *P* = 0.0426; 0.154 vs. 0.040%, *P* = 0.0104; Figures 2A,B) and HC group (0.219 vs. 0.072%, *P* = 0.0133; 0.154 vs. 0.049%, *P* = 0.0340; Figures 2A,B). We further analyzed the correlation between PD-1 expression and BATF expression of T cells in ATB patients. As expected, we found a significant positive correlation between PD-1 expression and BATF expression of T cells (for CD4^+^ T cells, *r* = 0.6761, *P* = 0.0158, Figure 2C; for CD8^+^ T cells, *r* = 0.6104, *P* = 0.0350, Figure 2D). These results suggested that BATF might play an inhibitory role in chronic TB infection, which may be correlated with PD-1.

### The Influence of PD-1/PD-Ls Pathway on the Expression of BATF in Patients With ATB

**Figure 3:**
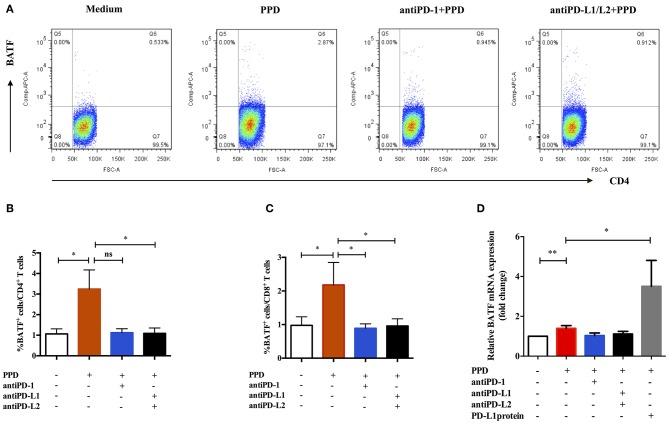
Expression of BATF was influenced by PD-1/PD-Ls pathway in patients with ATB. **(A)** Representative CD4-gated dot plots of BATF expression after blockade of PD-1/PD-Ls pathway. **(B)** BATF expressions in CD4^+^ T cells measured by flow cytometry after blockade of PD-1 or PD-L1/L2 (*n* = 9). **(C)** BATF expressions in CD8^+^ T cells measured by flow cytometry after blockade of PD-1 or PD-L1/L2 (*n* = 9). **(D)** Relative BATF mRNA expressions in PBMCs measured by real-time quantitative PCR after blockade of PD-1 or PD-L1/L2 or activation of PD-L1 (*n* = 11). Data are expressed as mean ± SEM. ^*^P < 0.05, ^**^P < 0.01.

We next tested whether intervention with PD-1/PD-Ls pathway could influence the expression of BATF in patients with ATB. To further understand the relationship of PD-1/PD-Ls pathway and BATF, we detected the effect of blockade of PD-1/PD-Ls pathway on expression of BATF using flow cytometry. The representative dot plots were shown in Figure 3A. As shown in Figures 3B,C, the BATF expressions of CD4^+^ T and CD8^+^ T cells increased after TB-specific antigen PPD stimulation (0.75 vs. 2.84%, *P* = 0.048 and 0.76 vs. 1.48%, *P* = 0.039, respectively), and decreased after blockade of PD-1 ligands with PD-L1/L2 blocking Abs (2.84 vs. 0.89%, *P* = 0.023 and 1.48 vs. 1.01%, *P* = 0.026, respectively). Interestingly, we observed an evident decrease of BATF expression in CD8^+^ T cells after blockade of PD-1 using PD-1 blocking Ab (1.48 vs. 0.85%, *P* = 0.014, Figure 3C), although no significant difference existed in BATF expression of CD4^+^ T cells, there was still a clear trending (2.84 vs. 1.17%, *P* = 0.0598, Figure 3B). To verify this result, we used real-time PCR to investigate the effect of PD-1/PD-Ls pathway on BATF mRNA level in PBMCs. We activated PD-1/PD-Ls pathway with human PD-L1 fusion protein, and found a significant elevation of BATF mRNA level in PBMCs after PD-L1 fusion protein combined stimulation compared with PPD alone stimulation (1.40 vs. 3.50, *P* = 0.0192, Figure 3D). Notably, the decreasing tendency of BATF mRNA level in PBMCs after blocking PD-1 ligation was not significantly different (Figure 3D). These results indicated that BATF expression could be induced by PD-1/PD-Ls pathway.

### Downregulation of BATF Enhanced the Cytokine Secretion of PPD-Specific PBMCs *in vitro*

**Figure 4:**
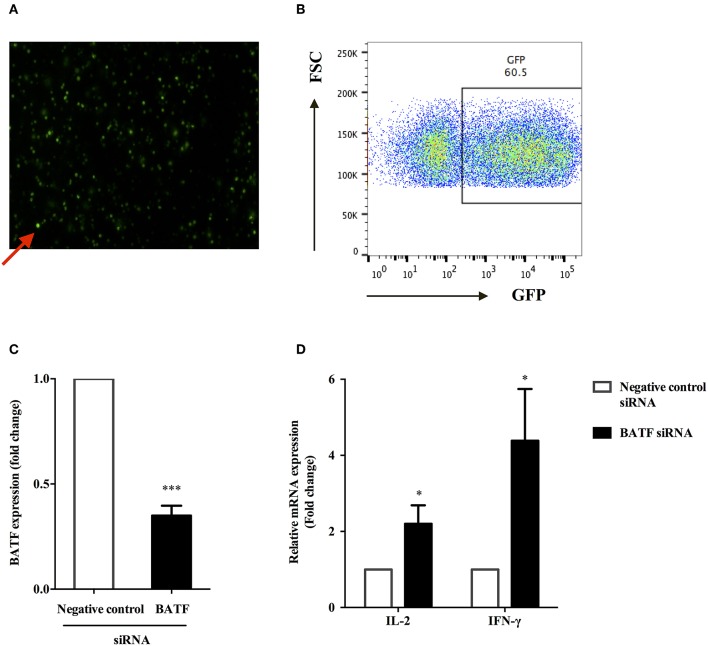
BATF silencing enhanced cytokine secretions by PPD-specific T cells in PBMCs in patients with ATB. **(A)** Representative fluorescence microscopy image of transfected primary human PBMCs after 24 h of electroporation with 2 μg GFP Vector, and red arrow indicated transfected PBMCs containing GFP (× 200). **(B)** Transfection efficiency of primary human PBMCs 24 h post electroporation were assessed by analysis of GFP protein expression by flow cytometry. **(C)** Silencing of BATF by a BATF siRNA SMART pool in primary human PBMCs from ATB patients measured by real-time quantitative PCR. Expression normalized to a housekeeping gene (*GAPDH*) is presented as fold change relative to negative control siRNA. **(D)** PBMCs were electroporated with indicated siRNA, then cultured with *M. tb*-specific antigen PPD (25 μg/mL) overnight, and IL-2 or IFN-γ mRNA levels were measured by real-time quantitative PCR. The information of controls and cell viability after transfection was provided in the Methods section BATF Small Interfering RNA (siRNA) Knockdown in ATB Patients (11). Data are expressed as mean ± SEM (*n* = 7). ^*^P < 0.05, ^***^P < 0.001. PPD, purified protein derivative; PBMCs, peripheral blood mononuclear cells; ATB, active tuberculosis; siRNA, small interfering RNA.

We sought to determine whether BATF could inhibit T cell function in patients with ATB. Therefore, we assessed PPD-specific PBMCs function after knocking down BATF (Figures 4A,B) by measuring cytokine secretion in response to *M. tb*-specific antigen PPD stimulation. The efficacy of siRNA uptake in primary human PBMCs after electroporation was assessed by detecting GFP fluorescence expression using flow cytometry. As shown in Figure 4A, red arrow indicated transfected PBMCs (green fluorescence), and the efficiency of electroporation was 60.5% (Figure 4B), which was consistent with the introduction of the standard Human T cell Nucleofector Kit. The extent of BATF knockdown in primary human PBMCs transfected with BATF siRNA SMART pool was confirmed by quantitative PCR. Compared with negative control siRNA group, BATF siRNA group remarkably decreased BATF mRNA level in PBMCs by an average of 65% (2^−ΔΔ*Ct*^ = 1.0 vs. 0.35, *P* < 0.001, Figure 4C). We further found that downregulation of BATF *in vitro* caused a significant increase in PPD-specific IL-2 and IFN-γ mRNA levels compared with negative control siRNA group (*P* = 0.0485 and *P* = 0.0473, respectively, Figure 4D). These results suggested that downregulation of BATF expression *in vitro* could enhance cytokine secretions of PBMCs in patients with ATB.

### BATF Silencing Improved the Proliferation of PPD-Specific CD4^+^ T Cells *in vitro*

**Figure 5:**
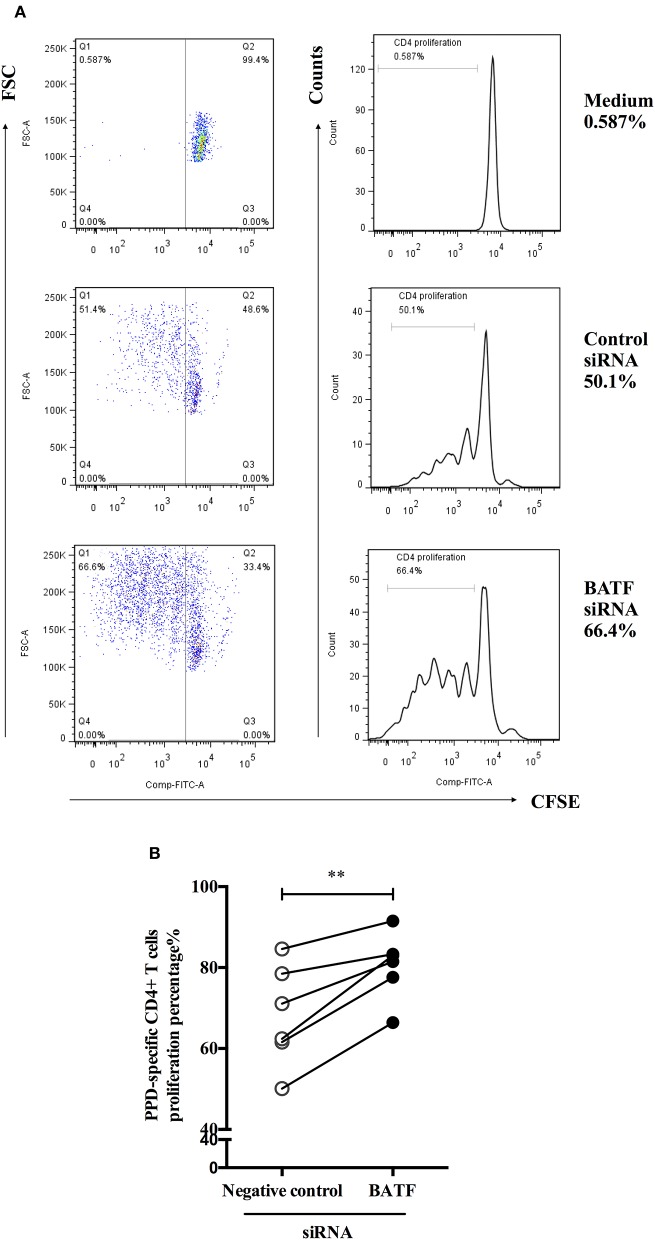
BATF silencing enhanced PPD-specific CD4^+^ T cells proliferation in patients with ATB *in vitro*. **(A)** Representative CD4-gated dot plots (left panel) and histograms (right panel) of proliferation of CFSE-labeled cells were measured by the fraction of CFSE^dim^CD4^+^ cells in the presence of PPD for 4 days after transfection. **(B)** PPD-specific CD4^+^ T cells proliferation in the BATF siRNA group was markedly higher than that in the negative control siRNA group (*n* = 6). Data are expressed as before-and-after plots. ^**^P < 0.01. PPD, purified protein derivative; ATB, active tuberculosis; siRNA, small interfering RNA.

To define the influence of BATF silencing on proliferation of CD4^+^ T cells from ATB patients in response to *M. tb*-specific antigen PPD *in vitro*. We further used a CFSE diluted assay to test proliferation of PPD-specific CD4^+^ T cells measured by the fraction of CFSE^dim^CD4^+^cells 4 days via flow cytometry after electroporation and PPD stimulation of PBMCs. As shown in Figure 5A, the representative dot plots (left panel) and histograms (right panel) of proliferation post transfection were presented. As expected, we found a significant increase in PPD-specific CD4^+^ T cells proliferation after BATF knockdown compared with negative control (68.05 vs. 80.55%, *P* = 0.0041, Figure 5B). Therefore, downregulating BATF expression could improve T cell function suppressed by co-inhibitory molecules in patients with ATB, including cytokine secretion and proliferation.

### BATF-Mediated PD-1/PD-Ls Pathway Inhibited Cytokine Secretion of PBMCs in Patients With ATB *in vitro*

**Figure 6:**
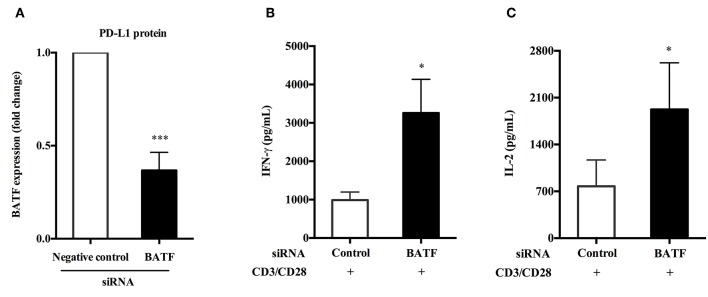
BATF-mediated PD-1/PD-Ls pathway inhibited cytokine secretion of PBMCs in patients with ATB. **(A)** After upregulation of BATF expression induced by human PD-L1 fusion protein, silencing of BATF by transfecting siRNA in primary human PBMCs from ATB patients measured by real-time quantitative PCR. Expression normalized to a housekeeping gene (*GAPDH*) is presented as fold change relative to negative control siRNA (*n* = 7). **(B,C)** Primary human PBMCs upregulated BATF expression were electroporated with indicated siRNA then cultured with anti-CD3 and anti-CD28 stimulation for 24 h, and IFN-γ **(B)** or IL-2 **(C)** protein levels in culture supernatants were measured by ELISA. Data are expressed as mean ± SEM (*n* = 7). ^*^P < 0.05, ^***^P < 0.001. PBMCs, peripheral blood mononuclear cells; ATB, active tuberculosis; siRNA, small interfering RNA.

We have previously confirmed that human PD-L1 fusion protein could upregulate the expression of BATF in primary human PBMCs compared with PPD alone stimulation (*P* = 0.0192, Figure 3D). Furthermore, silencing of BATF enhanced cytokine secretion and proliferation of T cells in ATB patients *in vitro*. Therefore, we hypothesized that PD-1/PD-Ls pathway played an immunosuppressive role in *M.tb* infection by inducing the expression of BATF that inhibit T cell function. To test this hypothesis, we first induced BATF upregulation with PD-L1 fusion protein described above, and further downregulated BATF expression by transfecting with BATF siRNA, finally we measured the cytokine secretion of PBMCs from ATB patients in response to antiCD3/CD28. As shown in Figure 6A, downregulation of BATF expression was determined by real-time quantitative PCR (*P* = 0.0006). Interestingly, we observed a significant increase in IFN-γ (988.4 vs. 3260 pg/mL, *P* = 0.0326, Figure 6B) and IL-2 (775.7 vs. 1925 pg/mL, *P* = 0.0317, Figure 6C) secretions in culture supernatants with anti-CD3 and anti-CD28 stimulation after BATF knockdown. These data suggested that BATF could mediate PD-1/PD-Ls pathway to inhibit cytokine secretion of PBMCs in patients with ATB.

## Discussion

Previous studies have confirmed that PD-1/PD-Ls pathway is correlated with impaired T cell function, which is involved in immunopathogenesis of TB (6–8, 10). The expressions of PD-1 and its ligands on the surface of T, B cells and monocytes were significantly upregulated in ATB patients (7, 9). Notably, our study also found that blockade of PD-1/PD-Ls pathway could partly restore *M. tb*-specific CD4^+^ T cell functions including IL-2, TNF-α and IFN-γ secretions and cells proliferation *in vitro* (7). PD-1 is known to inhibit T cell function by impairing TCR-mediated downstream signaling (25, 26). However, it is still unknown whether activation of PD-1/PD-Ls pathway is able to upregulate several genes impairing T cell function. In 2010, Quigley et al. observed enforced expression of BATF in exhausted HIV-specific CD8^+^ T cells and upregulation of BATF induced by PD-1/PD-Ls pathway, and found partly restoration of PD-1-mediated T cells exhaustion by silencing BATF (11). Thus, we reasoned that PD-1 upregulates the gene *BATF* in T cells which is involved in the inhibition of T cell function in the context TB. Here, we investigated the role of BATF in *M. tb* infection.

Few studies on BATF in tuberculosis were reported. A clinical study analyzed 7 cases of ATB and 8 cases of LTBI and found that gene *BATF* was much lower in ATB patients than LTBI cases, suggesting that *BATF* may provide new insights for the diagnosis of tuberculosis (27). However, in contrast to the results obtained in this study, we first enrolled three populations with different *M. tb* infection statuses including 26 ATB patients, 16 cases of LTBI and 22 HCs, and determined the expression of BATF in the peripheral blood using flow cytometry. We observed a significant increase in BATF expression of CD4^+^ and CD8^+^ T cells among ATB patients compared with LTBI and HC. The reasons for inconsistent results may be: (1) the former had a small sample size and had not been specifically described for the inclusion criteria of the study subjects, which might cause some selective bias; (2) the former determined the gene level of *BATF*, whereas we detected the protein level of BATF. Therefore, the role of BATF in tuberculosis deserves further investigation. Another study has shown that murine bone marrow-derived macrophages (BMDMs) stimulated with *M. tb* Beijing strain HN878 could induce the increased expression of BATF and BATF2 (21). This result demonstrated that *M. tb* infection could upregulate the expression of BATF, consistent with the result of this study.

We further found that the proportions of BATF^+^PD-1^+^ cells in both CD4^+^ and CD8^+^ T cells were much higher in ATB patients than those in LTBI and HC. It is conventionally believed that CD4^+^ T cells-mediated Th1 immune response plays a major role in fighting against TB, whereas *M. tb*-specific CD8^+^ T cells were detected in 60% of ATB and 15% of LTBI among a total of 326 cases (28). Furthermore, impaired *M. tb*-specific TNF-α and IFN-γ secretions in both CD4^+^ and CD8^+^ T cells were associated with the formation of pulmonary cavities (29). In acute and chronic mouse model of LCMV, it was observed that BATF could be expressed for a long time during chronic infection and believed that BATF could be used as a novel marker for exhausted CD8^+^ T cells (30, 31). This result was consistent with the study of Quigley et al. who found that PD-1 specifically induced the expression of BATF in HIV-specific CD8^+^ T cells and that overexpression of BATF in activated T cells reduced IL-2 secretion and cell proliferation *in vitro*, which are characteristics of exhausted T cells (11). Notably, we also found that the expression of BATF was significantly increased with increasing PD-1 expression on the surface of T cells in the context tuberculosis. These results indicated an inhibitory role of BATF on *M. tb* infection.

Given that the interaction of PD-1 and PD-L1 induced upregulation of HIV-specific CD8^+^ T cells BATF mRNA level, we reason that BATF mediates PD-1/PD-Ls pathway to play an immunosuppressive role in *M. tb* infection. To test this hypothesis, we investigated the effect of PD-1/PD-Ls pathway on BATF expression by either blocking or activating this pathway. As expected, we found that human PD-L1 fusion protein could activate PD-1/PD-Ls pathway to upregulate BATF mRNA expression in the presence of PPD stimulation, which is consistent with the study of HIV infection showing a two- to three-fold increase of BATF mRNA level in primary human T cells after culture with PD-L1, anti-CD3 and anti-CD28 (11). There was no significant difference in BATF expression after blockade of PD-1 in the mouse model of LCMV, but this study was lack of data about blockade of PD-1 ligands. Therefore, we further explored the influence of blockade of PD-1/PD-Ls pathway on BATF expression, and observed a significant decrease in BATF expression of both CD4^+^ and CD8^+^ T cells using flow cytometry after blockade of this pathway, especially when blocking PD-1 ligands.

BATF forms a Jun/BATF heterodimer by interacting with Jun protein rather than Jun/Fos heterodimer binding to DNA, thereby inhibiting AP-1-mediated function in immune response (12–14). Surprisingly, our results demonstrated that silencing BATF could enhance PPD-specific IL-2 and IFN-γ mRNA levels, and improve PPD-specific CD4^+^ T cells proliferation *in vitro*. Our findings were consistent with the data of HIV study, which reported that downregulation of BATF by shRNA or siRNA in both Jurkat cells and primary human T cells could increase IL-2 and IFN-γ secretion and CD8^+^ T cells proliferation *in vitro*. However, the study of HIV infection was absent of the data for simultaneous intervention with PD-1/PD-Ls pathway and BATF. We thereby activated PD-1/PD-Ls pathway with human PD-L1 fusion protein and downregulated BATF expression by transfection with siRNA, then we found a significant increase in IL-2 and IFN-γ secretion from culture supernatants in response to anti-CD3 and anti-CD28 stimulation. These results suggested that BATF knockdown partially reversed T cells dysfunction in context of *M. tb* infection. However, it is not clear that the detailed mechanism by which PD-1/PD-Ls pathway regulates BATF expression, or whether this alteration is directly or indirectly. The detailed mechanism may be further explored.

In conclusion, PD-1/PD-Ls pathway may mediate inhibition of T cell function in patients with ATB not only by impairing TCR signaling but also by upregulating the expression of BATF. BATF knockdown partially improves the cytokine secretion and cells proliferation *in vitro*, which may be a potential therapeutic target for anti-TB immunotherapy.

## Ethics Statement

This study was approved by the Ethics committee of Huashan Hospital, Fudan University. And written informed consent was obtained in accordance with the Declaration of Helsinki.

## Author Contributions

LSha, YG, and WZ conceived and designed the study. QL performed the experiments and drafted the manuscript. QO, LShe, and BZ contributed as clinicians. CQ and ZC participated in experimental guidance and data analysis.

### Conflict of Interest

The authors declare that the research was conducted in the absence of any commercial or financial relationships that could be construed as a potential conflict of interest.
