# Supplementary material for: BATF Potentially Mediates Negative Regulation of PD-1/PD-Ls Pathway on T Cell Functions in Mycobacterium tuberculosis Infection
Source: Front Immunol. 2019 Oct 15;10:2430. doi: 10.3389/fimmu.2019.02430 (PMC6803382; doi:10.3389/fimmu.2019.02430)
Supplement: Supplementary file 1 [file Data_Sheet_1.docx]

**Supplementary materials**


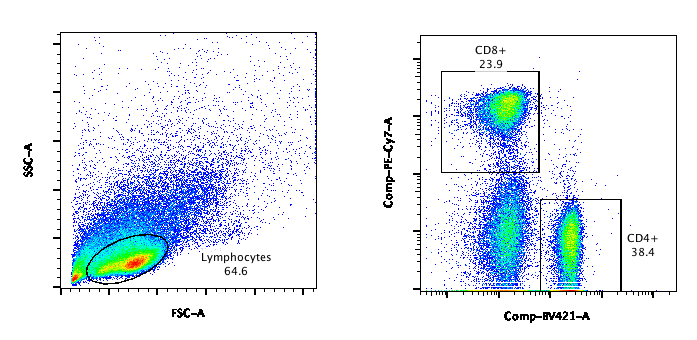


**Figure S1.** Gating strategy to identify CD4^+^ and CD8^+^ T cells in a representative case.


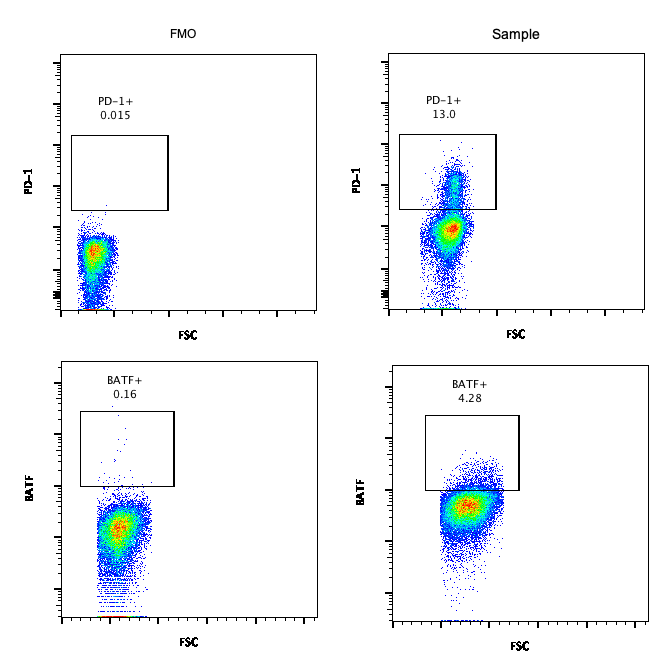


**Figure S2.** Gating strategy to identify PD-1^+^ and BATF^+^ T cells using FMO controls in a representative case. FMO, fluorescence minus one.

**
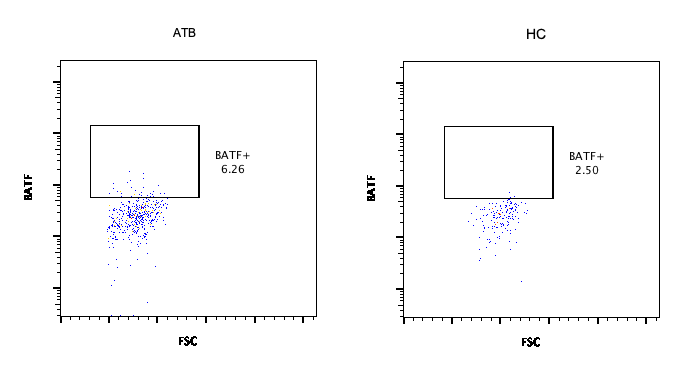
**

**Figure S3.** BATF expression on PD-1^+^ T cells in a representative case with ATB compared with HC gated from PD-1^+^ cells. ATB, active tuberculosis; HC, healthy control.
